# Human Extravillous Trophoblasts Penetrate Decidual Veins and Lymphatics before Remodeling Spiral Arteries during Early Pregnancy

**DOI:** 10.1371/journal.pone.0169849

**Published:** 2017-01-12

**Authors:** Nannan He, Liesbeth van Iperen, Danielle de Jong, Karoly Szuhai, Frans M. Helmerhorst, Lucette A. J. van der Westerlaken, Susana M. Chuva de Sousa Lopes

**Affiliations:** 1 Department of Anatomy and Embryology, Leiden University Medical Center, Leiden, The Netherlands; 2 Department of Molecular Cell Biology, Leiden University Medical Center, Leiden, The Netherlands; 3 Department of Gynaecology, Division of Reproductive Medicine, Leiden University Medical Center, Leiden, The Netherlands; 4 Department for Reproductive Medicine, Ghent University Hospital, Ghent, Belgium; VU medisch centrum, NETHERLANDS

## Abstract

In humans, the defective invasion of the maternal endometrium by fetal extravillous trophoblasts (EVTs) can lead to insufficient perfusion of the placenta, resulting in pregnancy complications that can put both mother and baby at risk. To study the invasion of maternal endometrium between (W)5.5–12 weeks of gestation by EVTs, we combined fluorescence in situ hybridization, immunofluorescence and immunohistochemistry to determine the presence of (male) EVTs in the vasculature of the maternal decidua. We observed that interstitial mononuclear EVTs directly entered decidual veins and lymphatics from W5.5. This invasion of decidual veins and lymphatics occurred long before endovascular EVTs remodelled decidual spiral arteries. This unexpected early entrance of interstitial mononuclear EVTs in the maternal circulation does not seem to contribute to the materno-placental vascular connection directly, but rather to establish (and expand) the materno-fetal interface through an alternative vascular route.

## Introduction

During human placental development, at the tips of the anchoring villi, cytotrophoblasts proliferate into cell columns and from there they can detach and invade the maternal decidualized endometrium (decidua) and even the myometrium [1, 2]. Those invading fetal cytotrophoblasts are known as extravillous trophoblasts (EVTs).

At term, there are several subtypes of EVTs present in the maternal decidua, depending on their localization: the interstitial mononuclear (as well as multinuclear) EVTs are dispersed in the decidual mesenchyme; the endovascular EVTs and intramural EVTs are both directly associated with remodeled spiral arteries and are present in their lumen (or replacing the endothelial cells) and in their tunica media [3] respectively and those EVTs migrate-colonize the spiral arteries in a retrograde fashion [4]; and a fourth category of ‘epithelial’ EVTs lines, together with maternal endothelial cells in a mosaic fashion, the basal plate of the maternal decidua basalis [5].

In humans, the defective invasion of the maternal decidua by fetal EVTs leads to insufficient perfusion of the placenta, resulting in pregnancy complications, such as intrauterine growth restriction, (recurrent) spontaneous abortion, (very) premature birth and preeclampsia [6, 7]. The unique vascular remodeling of the maternal decidua during pregnancy, whereby both fetal and maternal cells play complementary regulatory roles, is a fundamental process for a successful pregnancy [8, 9].

Endovascular EVTs are observed in spiral arteries from (W)8 weeks of gestation, ‘plugging’ (or blocking) the entrance of spiral arteries to the intervillous space preventing maternal blood flow until about W12 [10–12]. During this period (W8-W12), decidual veins become dilated in both decidua parietalis and basalis to give rise to decidual venous lakes [13]. By W12, both decidual veins and spiral arteries are in open connection with the intervillous space of the placenta allowing the maternal blood to circulate between the placental villi [10–12]. This materno-placental vascular connection provides efficient exchange of nutrients, waste products, hormones and gases between the maternal blood and the fetal blood and is crucial to ensure fetal growth.

After the materno-placental vascular connection is established, the constant maternal blood flow transports small tissue fragments (syncytial knots) that are shed from the outer layer of the placental villi of multinucleated (non-viable) syncytiotrophoblasts into the decidual venous where those knots become trapped and are subsequently cleared [13–15]. If the syncytial knots escape to enter the maternal circulation, they can cause trophoblastic embolism and even sudden death of the mother [16].

Interestingly, the presence of fetal cells [17], placental-derived particles and exosomes [18, 19] in the maternal blood from W6 has been described, but in the absence of robust materno-placental vascular connection this remains to be clarified. Therefore, we sought to systematically characterize the presence of EVTs in the endometrium in the period W5.5-W12. Our results indicate that between W5.5-W12, interstitial mononuclear EVTs efficiently enter the maternal (blood and lymph) circulation. This novel aspect of EVT invasion, enlarging materno-fetal interface early during pregnancy, may shed new light in our understanding of pregnancy complications and maternal immune tolerance.

## Materials and Methods

### Ethics approval and human tissue collection

The Medical Ethics Committee of the Leiden University Medical Center (protocol P08.087) approved the collection and use of material for this study. Written informed consent was obtained from all patients (N = 13). Tissue was obtained by vacuum aspiration from women undergoing voluntary pregnancy termination without medical indication and obtained anonymized. The gestational age (in weeks and days) was determined by obstetric ultrasonography and can be converted to weeks post conception by subtracting two weeks. Tissue samples (S1 Table) were collected in cold saline solution (0.9% NaCl). The decidua basalis and parietalis were identified retrospectively by the presence of scattered pKRT-positive EVTs in histological sections.

### Sex genotyping and histochemistry

The material was sex genotyped by polymerase chain reaction (PCR) for AMELX/AMELY as previously described [20]. The decidua was fixed in 4% paraformaldehyde (PFA) overnight at 4°C, paraffin embedded and sectioned as previously described [20]. For Azan staining, paraffin sections were deparaffinised using xylene and dehydrated by standard procedures. The sections were then treated with 0.1% Azocarmine B (Edward Gurr Ltd., London, UK) in 5% glacial acetic acid (Merck, Darmstadt, Germany) for 3 minutes, rinsed several times in water, treated with 5% phosphotungstic acid (Fluka, Sigma-Aldrich, St. Louis, USA) solution for 6 minutes, again rinsed several times in water and immersed in a 1:1 solution of water to 0.4% orange G (Merck, Darmstadt, Germany)/ 0.2% aniline blue (Nustain, Nottingham, UK) in 1% glacial acetic acid for 15 minutes, all at room temperature (RT). Sections were then rinsed in water, dehydrated through a series of ethanol solutions, xylene and mounted in Entellan (Merck, Darmstadt, Germany).

### Immunofluorescence

Paraffin sections were deparaffinised using xylene and dehydrated by standard procedures and used for immunofluorescence as previously described [20]. Briefly, antigen retrieval was performed in citrate buffer, followed by treatment with blocking solution consisting of 1% bovine serum albumin (BSA; Sigma-Aldrich, St. Louis, USA) in 0.05% PBST [0.05% Tween-20 (Merck-Schuchardt, Hohenbrunn, Germany) in phosphate-buffered saline (PBS)] for 40 minutes at RT. Sections were then incubated with the primary antibodies diluted in blocking solution for 1 hour at RT, washed twice in PBST for 5 minutes and incubated with secondary antibodies diluted in blocking solution for 1 hour at RT. The primary antibodies used were mouse anti-pan-keratin (pKRT; 1:100; AE1/AE3, Dako, Heverlee, Belgium), rabbit anti-platelet/endothelial adhesion molecule 1 (PECAM1; 1:200; sc1506-R, Santa Cruz Biotechnology, Santa Cruz, CA, USA), rabbit anti-alpha-smooth muscle actin (ACTA2; 1:200; ab5694, Abcam, Cambridge, UK), mouse anti-podoplanin (PDPN; 1:100; ab77854, Abcam, Cambridge, UK) and rat anti-PDPN (1:100; 12-9381-42, eBioscience, San Diego, CA, USA). The secondary antibodies used were Alexa Fluor 594 donkey anti-mouse IgG (1:500; A-21203), Alexa Fluor 488 goat anti-rabbit IgG (1:500; A-11034), Alexa Fluor 555 goat anti-rat IgG (1:500; A-21434), Alexa Fluor 488 donkey anti-mouse IgG (1:500; A-21202) and Alexa Fluor 647 donkey anti-rabbit IgG (1:500; A-31573) (all from Life Technologies, Eugene, OR, USA). Nuclei were stained with 4′,6-diamidino-2-phenylindole (DAPI; Vector Laboratories Ltd., Peterborough, UK) and sections were mounted in Prolong Gold anti-fade reagent (Life technologies). As negative controls, slides were immunostained omitting the primary antibodies.

### Fluorescence in situ hybridization (FISH) for chrX and chrY

FISH with probes of human chrX and chrY was carried out to distinguish fetal (male) cells from maternal (female) cells. After selected immunostained slides were imaged, they were incubated with 0.4% pepsin (pepsin from porcine gastric mucosa, P7000-100G, Sigma-Aldrich, St Louis, MO, USA) in 0.02M HCl for 5 minutes at 37°C. Centromere specific alphoid repeat probes used were pBamX5 and pDP97 of the human chrX and chrY, respectively [21, 22]. Both probes were labeled by nick translation with FITC (chrX probe) or Cy3 (chrY probe). Probe labeling and hybridization reaction were performed using previously published protocols [23, 24].

### Image acquisition

Slides were either scanned on a Pannoramic 250 FLASH digital scanner (3D HISTECH Ltd., Budapest, Hungary) and representative areas selected for images using the software program ‘Pannoramic viewer’ (3D HISTECH Ltd., Budapest, Hungary); or photographed on a Leica DMRA fluorescence microscope (Leica, Wetzlar, Germany) equipped with a CoolSnap HQ2 camera (Photometrics, Tucson, USA). Figures were compiled using Photoshop CS6 (Adobe Systems Inc., San Jose, USA).

### Quantification of veins being invaded by EVTs

The combination of Azan staining and immunofluorescence for ACTA2, PECAM1 and PDPN on serial sections from the decidua basalis enabled us to distinguish decidual veins from arteries and lymphatic vessels. Decidual veins were PECAM1-positive, ACTA2-negative and PDPN-negative vessels, with reduced amounts of extracellular matrix surrounding them. Veins were considered invaded when there were one or multiple (male) EVTs (KRT-positive, chrY-positive) penetrating the (maternal) vessel wall (chrY-negative, PECAM1-positive, ACTA2-negative and PDPN-negative). To quantify the percentage of decidual veins invaded by EVTs, we manually counted decidual veins (with and without EVTs invasion) in each paraffin section of decidua basalis of different individuals (N = 9) between W5.5-W12. The number of paraffin sections (n) counted is shown. The results are shown as mean ± standard deviation.

## Results

### Interstitial mononuclear EVTs invade decidual veins from W5.5-W7.5

We investigated the presence of (male) EVTs in the decidua in the period W5.5-W12 and have analyzed a total of n = 19 samples of decidua basalis and n = 20 decidua parietalis from N = 13 women (S1 Table). Due to a paucity of markers to identify specifically EVTs, we used a combined approach of cytogenetics (FISH for sex chromosomes chrX and chrY) and immunofluorescence for pan-keratin (pKRT) to distinguish maternal glandular epithelial cells from the fetal (male) EVTs in the endometrium (S1 Fig). This staining-combination also allowed us to distinguish decidua parietalis from decidua basalis (S1 Fig). Combining cytochemistry (Azan staining) and immunofluorescence for the arterial-marker ACTA2, the endothelial-marker PECAM1 and the lymphatic-marker PDPN, we were able to differentiate between arteries, veins and lymphatic vessels.

**Fig 1:**
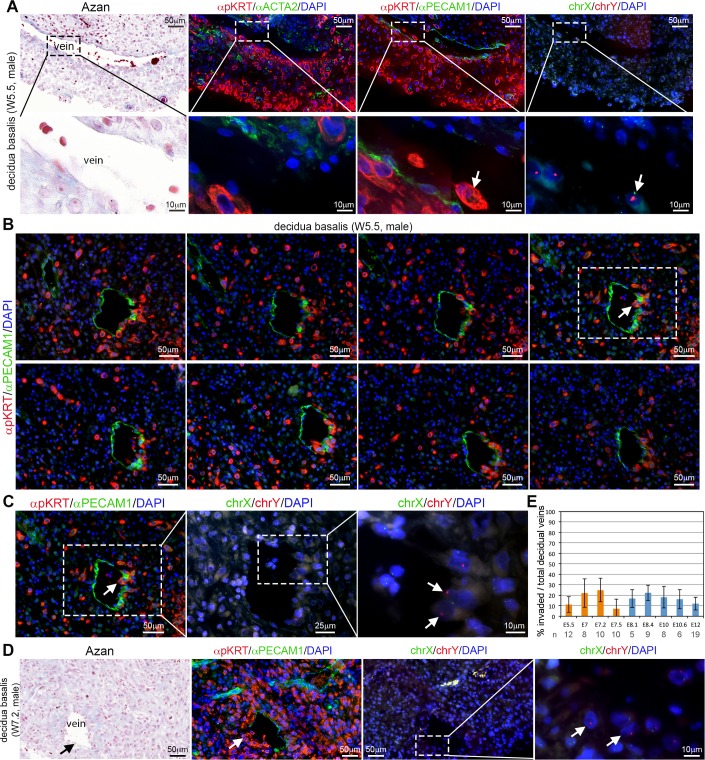
Human EVTs penetrate decidual veins early during first trimester (W5.5-W7.5). (A) Histological sections of decidua basalis at W5.5 used for Azan staining (left panel), immunostained for pKRT and ACTA2 (left-middle panel) and immunostained for pKRT and PECAM1 (right-middle panel). FISH for chrX/chrY (right) was performed in the pKRT/PECAM1-stained sections. White arrows indicate male EVTs invading veins. The bottom row shows magnifications of the dashed boxes in the top row. (B) Consecutive sections of W5.5 decidua basalis were immunostained for pKRT/PECAM1. (C) FISH for chrX and chrY (right panels) magnifications are shown for the section in dashed box. White arrows point to male EVTs penetrating a decidual vein. (D) Histological sections of decidua basalis at W7.2 used for Azan staining (left panel), immunostained for pKRT and PECAM1 (left-middle panel). FISH for chrX/chrY (right panels) was performed in the pKRT/PECAM1-stained sections. The most right panel shows a magnification of the dashed box. White arrows indicate male EVTs invading veins. (E) Percentage of invaded decidual veins per total veins encountered per histological section (n) between W5.5-W12. Results are sown as mean ± standard deviation. All scale bars are depicted.

Surprisingly, between W5.5-W7.5 in the decidua basalis, we detected invasion of (non-dilated) ACTA2-negative PECAM1-positive decidual (maternal) veins by (male) individual interstitial mononuclear EVTs (Fig 1A–1E). These (male) EVTs were clearly observed penetrating the endothelial wall of decidual veins, often in consecutive sections (Fig 1B) in all the W5.5-W7.5 decidua basalis analyzed (n = 9 from N = 6 individuals). We counted decidual veins in paraffin sections (n = 40) from different individuals between W5.5-W7.5 (N = 4), and observed that on average 17% (147/856) of the decidual veins were invaded by EVTs (Fig 1E, orange bars).

**Fig 2:**
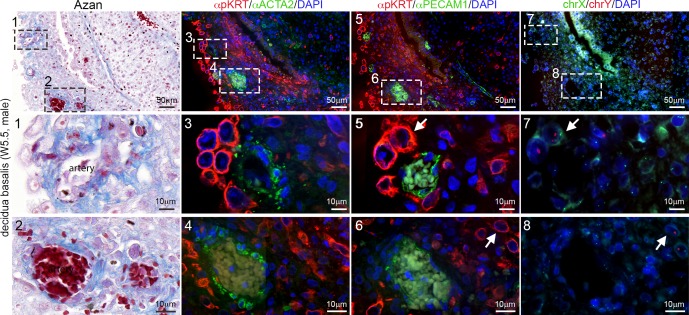
Human EVTs in the vicinity of arteries early during first trimester (W5.5). Histological sections of W5.5 decidua basalis showing decidual arteries. Consecutive sections were used for Azan staining (left panel), immunostained for pKRT and ACTA2 (left-middle panel) and immunostained for pKRT and PECAM1 (right-middle panel). FISH for chrX and chrY (right panel) was performed in the pKRT/PECAM1-stained sections. The (middle and) bottom rows show magnifications of the (numbered) dashed boxes in the top rows. White arrows depict male EVTs. All scale bars are depicted.

By contrast, individual interstitial mononuclear EVTs were always observed outside the arterial smooth-muscle layer of the ACTA2-positive PECAM1-positive decidual spiral arteries, even in those arteries located close to the maternal basal plate and in the vicinity of EVTs (Fig 2).

### From W8-W12, mononuclear EVTs invade spiral arteries as well as decidual veins

**Fig 3:**
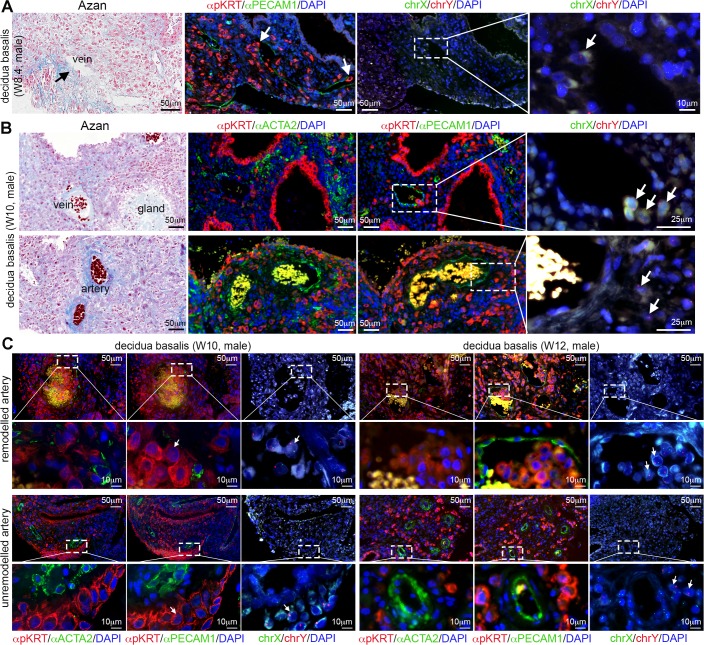
Human extravillous trophoblast cells in decidua between W8-W12. (A) Histological sections of decidua basalis at W8.4 used for Azan staining (left panel), immunostained for pKRT and PECAM1 (left-middle panel). FISH for chrX/chrY (right panels) was performed in the pKRT/PECAM1-stained sections. The most right panel shows a magnification of the dashed box. White arrows indicate male EVTs invading veins. (B) Histological sections of decidua basalis at W10 used for Azan staining (left panel), immunostained for pKRT and ACTA2 (left-middle panel) and immunostained for pKRT and PECAM1 (right-middle panel). FISH for chrX/chrY (right) was performed in the pKRT/PECAM1-stained sections. White arrows indicate male EVTs. Low magnifications are shown in S2 Fig. (C) Histological sections of W10 and W12 decidua basalis showing remodeled and unremodelled arteries. Consecutive sections were immunostained for pKRT and ACTA2 (left panels) and immunostained for pKRT and PECAM1 (middle panels). FISH for chrX and chrY (right panels) was performed in the pKRT/PECAM1-stained sections. The bottom rows show magnifications of the dashed boxes in the top rows. All scale bars are depicted.

Between W8-W12 (N = 7), interstitial mononuclear EVTs were still observed penetrating many decidual veins both close to the basal plate and more interiorly located (Fig 3A and 3B and S2 Fig). We counted decidual veins in paraffin sections (n = 47) from different individuals between W8.1-W12 (N = 5), and observed that on average 16% (165/1038) of the decidual veins were invaded by EVTs (Fig 1E, blue bars), comparable to what was observed between W5.5-W7.5. At this gestational age, many decidual spiral arteries located at the maternal basal plate were indeed being remodeled and contained endovascular and intramural EVTs in the lumen and tunica media, respectively (Fig 3C). However, we noticed that deeper located decidual spiral arteries remained unremodelled, whereas neighboring deeper located veins were consistently invaded by individual interstitial mononuclear EVTs in all decidua basalis analyzed (Fig 3B and 3C and S2 Fig).

**Fig 4:**
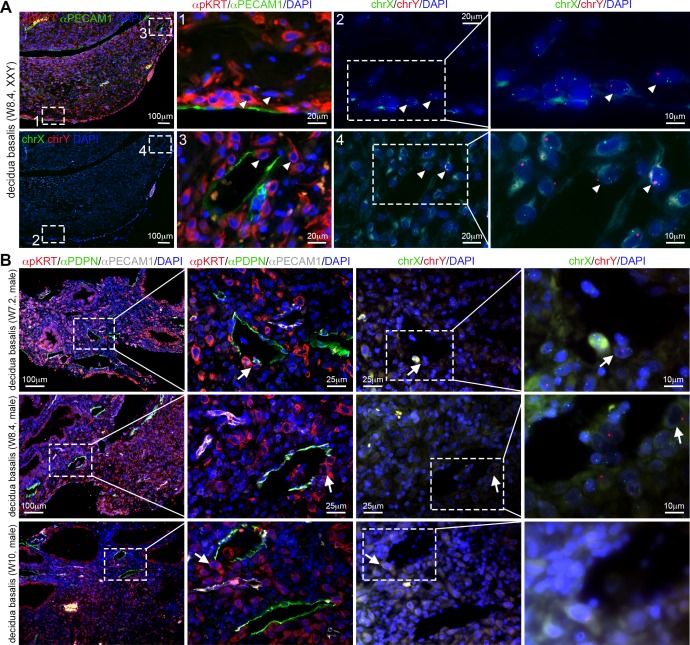
Human extravillous trophoblast cells invade decidual veins and lymphatics early during first trimester. (A) Histological sections of decidua basalis from a (mosaic) Klinefelter syndrome (47,XXY and 46, XY) pregnancy at W8.4. Left panels show low magnifications of same section immunostained for pKRT and PECAM1 (top) and chrX/chrY FISH (bottom). Middle-left and middle-right panels are magnifications of the (numbered) dashed boxes in the left panels. Right panels are magnifications of the dashed boxes in the middle-right panels. White arrowheads point to XXY fetal EVTs. (B) Histological sections of decidua basalis at W7.2, W8.4 and W10 immunostained for pKRT, PDPN and PECAM1 (left panels). FISH for chrX and chrY (right) was performed in the in the pKRT/PDPN/PECAM1-stained sections. White arrows point to EVTs invading the lymphatic vessels. All scale bars are depicted.

From the decidual samples analyzed in this study, cytogenetic analysis revealed that one individual decidua was from a W8.4 conceptus with Klinefelter syndrome (mosaic 47,XXY and 46,XY) (Fig 4A), even though additional chromosomal abnormalities were not investigated. Importantly, interstitial mononuclear XXY EVTs were observed entering decidual veins (Fig 4A), as in the other decidua of similar age, suggesting that this aspect of the establishment of the materno-fetal interface occurred normally.

### Interstitial mononuclear EVTs entered decidual lymphatics from W5.5-W12

To exclude that the PECAM1-positive ACTA2-negative vessels were lymphatic vessels instead of veins, we investigated the expression of PDPN in the decidua [25–27]. Notably, not only PDPN-negative PECAM1-positive decidual veins were being invaded by interstitial mononuclear EVTs, but also many PDPN-positive PECAM1-positive decidual lymphatic vessels were in fact being invaded by interstitial mononuclear EVTs between W5.5-W12 (N = 11 out of 12 analyzed) (Fig 4B). We observed dilated PDPN-positive lymphatics encircling (or insulating) spiral arteries to create characteristic round structures (data not shown), confirming findings by Volchek and colleagues (2010) [27]. The biological significance of these characteristic structures remains to be investigated. We also report PDPN-positive mesenchymal stroma inside the placental villi (cyan arrow in S2 Fig, top right panel), as previously reported by Wang et al (2011) [28].

### Aggregates of mononuclear EVTs were present in decidual veins from W5.5

**Fig 5:**
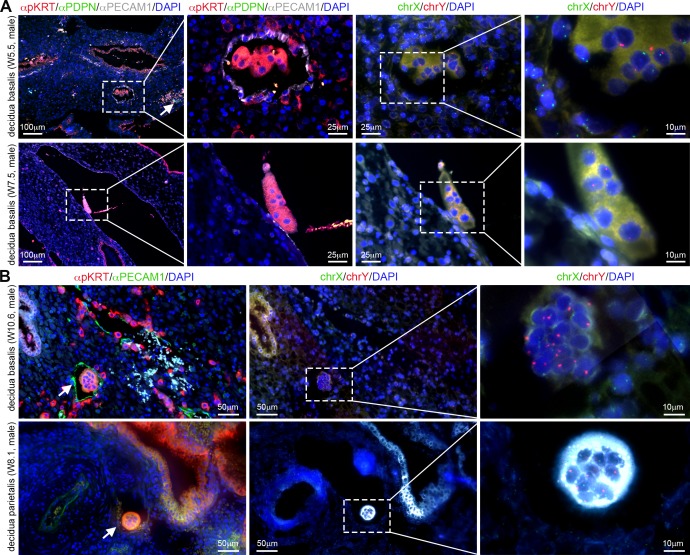
EVT aggregates and syncytial knots in the decidua basalis and parietalis. (A) Histological sections of decidua basalis at W5.5 and W7.5 immunostained for pKRT, PDPN and PECAM1 (lefts panels) showing the presence of mononuclear EVT aggregates (top row) and syncytial knots (bottom row). FISH for chrX and chrY (right panels) was performed in pKRT/PDPN/PECAM1-stained sections. White arrow indicates PDPN-positive lymphatics. (B) Histological sections of W10.6 decidua basalis (top row) and W8.1 decidua parietalis (bottom row) immunostained for pKRT and PECAM1 showing the presence of syncytial knots in veins. FISH for chrX and chrY (right panels) was performed in pKRT/PECAM1-stained sections. All scale bars are depicted.

At W5.5, we could already observe small aggregates of mononuclear EVTs in veins in the decidua basalis (Fig 5A). Those resembled syncytial knots, but were morphologically distinct. At later time points, from W7-W12 (N = 6), bona fide syncytial knots (central area of multiple nuclei and large peripheral area of cytoplasm) were often present in dilated veins in both the decidua basalis (Fig 5A and 5B) and parietalis (Fig 5B), suggestive of the gradual establishment of materno-placental vascular connection.

### Allo-epi-endothelium in the basal plate of first trimester decidua basalis

**Fig 6:**
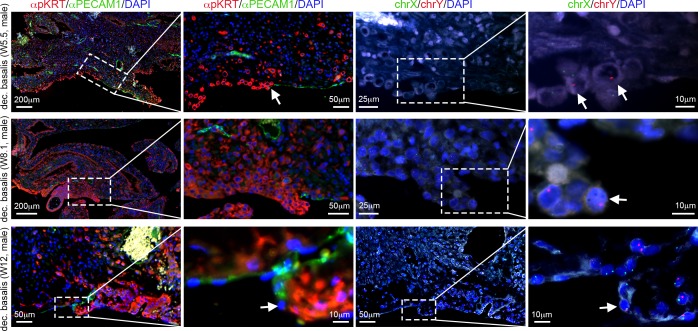
Allo-epi-endothelial surface of the decidua basalis during first trimester. Histological sections of decidua basalis containing the basal plate of the decidua basalis immunostained for pKRT and PECAM1 at W5.5, W8.1 and W12. FISH for chrX/chrY was also performed on the pKRT/PECAM1-stained sections. Magnifications of the dashed boxes are shown. White arrows point to EVTs bordering with maternal PECAM1-positive endothelial cells in the basal plate of the maternal decidua basalis. All scale bars are depicted.

Finally, we report that the basal plate of the maternal decidua (between the anchoring villi) was lined by both (male) pKRT-positive EVTs and (female) PECAM1-positive maternal endothelial cells in the period analyzed (W5.5-W12) (Fig 6). This allo-epi-endothelium monolayer organization of ‘epithelial’ EVTs and maternal endothelial cells in a patchy-mosaic fashion has been described in term decidua [5] and we describe it now in the human decidua during the first trimester as well.

## Discussion

### Interstitial mononuclear EVTs enter the maternal circulation via veins and lymphatics

Our systematic analysis of first trimester decidua using a combination of immunofluorescence and FISH to distinguish between the types of vasculature and identify the EVTs unambiguously allowed us to reveal for the first time the direct invasion by interstitial mononuclear EVTs of decidual veins and lymphatics in the maternal decidua basalis by W5.5. This suggests an efficient entry of interstitial mononuclear EVTs into the maternal (blood and lymph) circulation in the absence of robust materno-placental vascular connection [10–12].

Consistent with our findings, very recently the group of Huppertz has also showed evidence for EVTs invasion of uterine veins [29], using an antibody against human leukocyte antigen (HLA)-G, often used as specific EVT marker [30]. However, caution should be taken when using only HLA-G to identify EVTs, due to the different HLA-G isoforms [31]. In addition, using HLA-G immunostaining as means to identify EVTs, the same group has also reported that EVTs (coined “endoglandular”) also invaded uterine glands [29, 32, 33]. In our study, using a combination of immunofluorescence and FISH, we were unable to detect (male) EVTs in maternal uterine glands between W5.5 and W12.

The timing of the venous invasion process (by the EVTs) is different from the passive deportation of (non-viable) syncytial knots detaching directly from placental villi to the intervillous space and being then transported to the decidual venous lakes once the materno-placental vascular connection has been established [13–15, 34].

### Materno-fetal interface: EVTs stepping out of the uterus

The materno-fetal interface encompasses three main processes: the establishment of immune tolerance, regulation of decidual invasion by EVTs and uterine vascular remodeling [35], all taking place primarily in the uterus. Our results are in agreement with the timing of vascular remodeling as well as with the invasion of the decidua by interstitial and endovascular EVTs. Moreover, we observed the formation of the allo-epi-endothelium in the basal plate of the decidua basalis and therefore acknowledge the existence of epithelial EVTs [5].

Here, we propose that when interstitial mononuclear EVTs enter the maternal circulation they are not only substantially enlarging the materno-fetal interface, but they are also doing so earlier than previously accepted [via placental debris after materno-placental vascular connection [18]] and via an alternatively vascular route (decidual lymphatics and veins).

This novel venue of materno-fetal interface (taking place outside the uterus) may contribute significantly to trigger the maternal immune system to perhaps confer recognition to prevent rejection of the fetus while in the uterus. The EVTs have developed several strategies to ensure immune tolerance while in the maternal environment. This includes the expression of HLA-G, instead of HLA-A and HLA-B, in this way avoiding clearance by maternal natural killer cells [36]. In the uterus, the EVTs encounter and crosstalk with uterine macrophages, uterine natural killer cells and T lymphocytes, modulating locally the maternal innate and adaptive immune response [35, 36]. The EVTs that enter the maternal circulation may have a primary role contributing to the adaptive and innate immune response, but through the recruitment of maternal cells to the uterus subsequently also contribute to successful vascular remodeling [4].

### Materno-fetal interface and pregnancy complications

Sex chromosomal aneuploidy disorders, including Klinefelter syndrome, often produce small-for-gestational age fetuses [37]. We describe here the decidua basalis of one W8.4 pregnancy with Klinefelter syndrome (mosaic 47,XXY and 46,XY) and show interstitial mononuclear EVTs entering the maternal circulation via decidual veins. We were unable to detect “plugged” of remodeled arteries colonized by endovascular EVTs, probably due to the young age of the fetus.

Our understanding of placental complications in humans is rather limited due to the lack of *in vitro* assays and suitable animal models. However it is clear that the materno-fetal interface in all its facets is indispensable for a successful pregnancy [38, 39]. We speculate that pregnancy complications such as preeclampsia and intrauterine growth restriction that are closely associated to defects in vascular remodeling [3, 7, 40], as well as recurrent abortion, may correlate with defective presence of EVTs in the maternal circulation.

## Supporting Information

S1 Fig Distinguishing male embryonic EVTs from female maternal glandular epithelial cells. (A-B) Decidua basalis (A) and parietalis (B) immunostained for pKRT and ACTA2, followed by FISH for chromosome (chr) X and chrY in the pKRT/ACTA2-stained sections. The middle and bottom rows show magnifications of the (numbered) dashed boxes in the top row. White arrowheads depict female maternal glandular epithelial cells (gland cells), white arrows depict male EVTs and cyan arrows depict an unremodelled artery containing no (male) EVTs. All scale bars are depicted.
(TIF)

S2 Fig Human extravillous trophoblast cells in decidua at W10. Histological sections of W10 decidua basalis showing decidual veins, arteries and lymphatic vessels. Consecutive sections were used for Azan staining (left panels), immunostained for pKRT and PECAM1 (left-middle panels), immunostained for pKRT and ACTA2 (right-middle panels) and immunostained for PDPN and PECAM1 (right panels). The middle and bottom rows show magnifications of the (numbered) dashed boxes in the top rows. White arrow depicts EVTs invading a vein; cyan arrow points to an attached placental villus; white arrowheads point to a PDPN-positive lymphatic vessel. FISH for chrX and chrY is shown in Fig 3. All scale bars are depicted.
(TIF)

S1 Table Characteristics of the human decidua samples. (DOC)
