# Supplementary figures and images for: Human Extravillous Trophoblasts Penetrate Decidual Veins and Lymphatics before Remodeling Spiral Arteries during Early Pregnancy
Source: PLoS One. 2017 Jan 12;12(1):e0169849. doi: 10.1371/journal.pone.0169849 (PMC5230788; doi:10.1371/journal.pone.0169849)

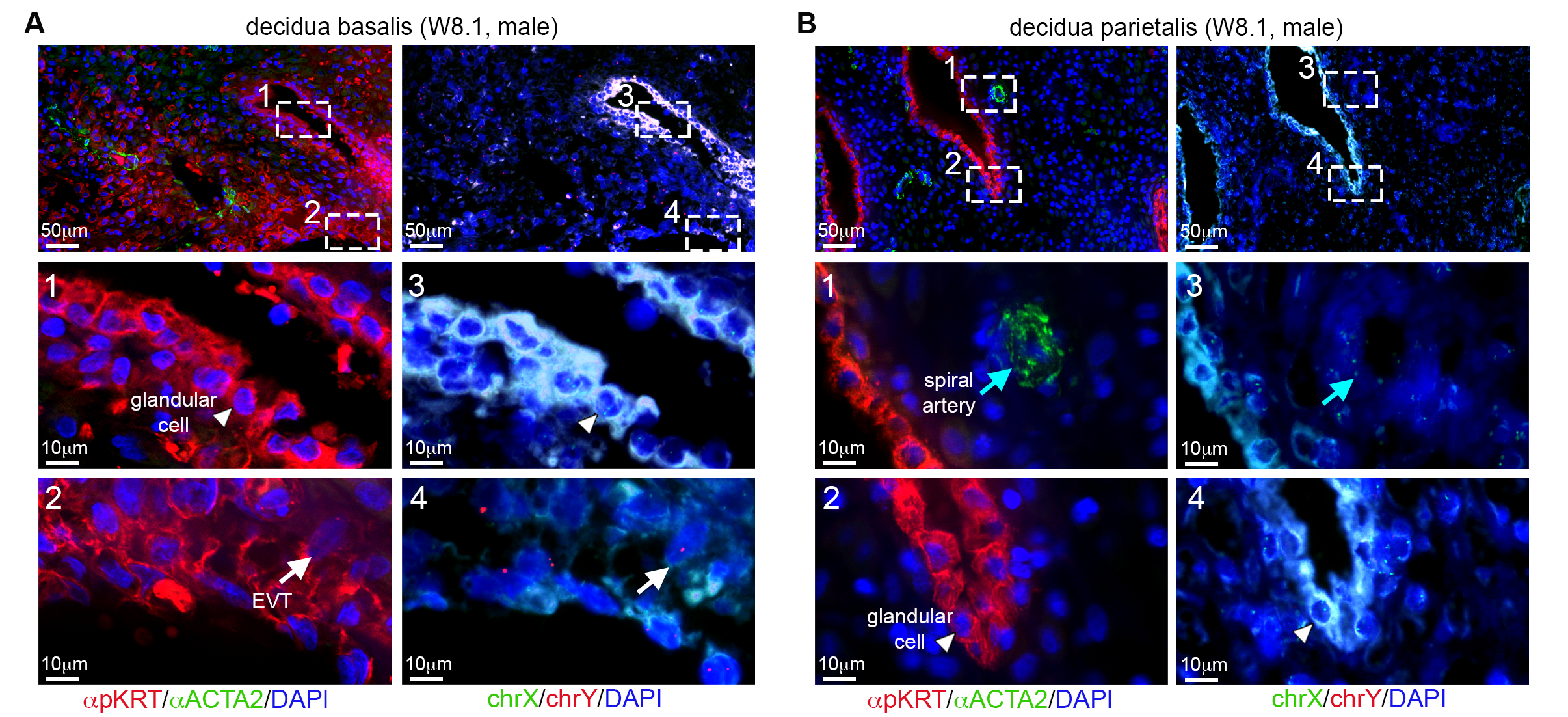

Supplement: S1 Fig — (A-B) Decidua basalis (A) and parietalis (B) immunostained for pKRT and ACTA2, followed by FISH for chromosome (chr) X and chrY in the pKRT/ACTA2-stained sections. The middle and bottom rows show magnifications of the (numbered) dashed boxes in the top row. White arrowheads depict female maternal glandular epithelial cells (gland cells), white arrows depict male EVTs and cyan arrows depict an unremodelled artery containing no (male) EVTs. All scale bars are depicted. (TIF) [file pone.0169849.s001.tif]

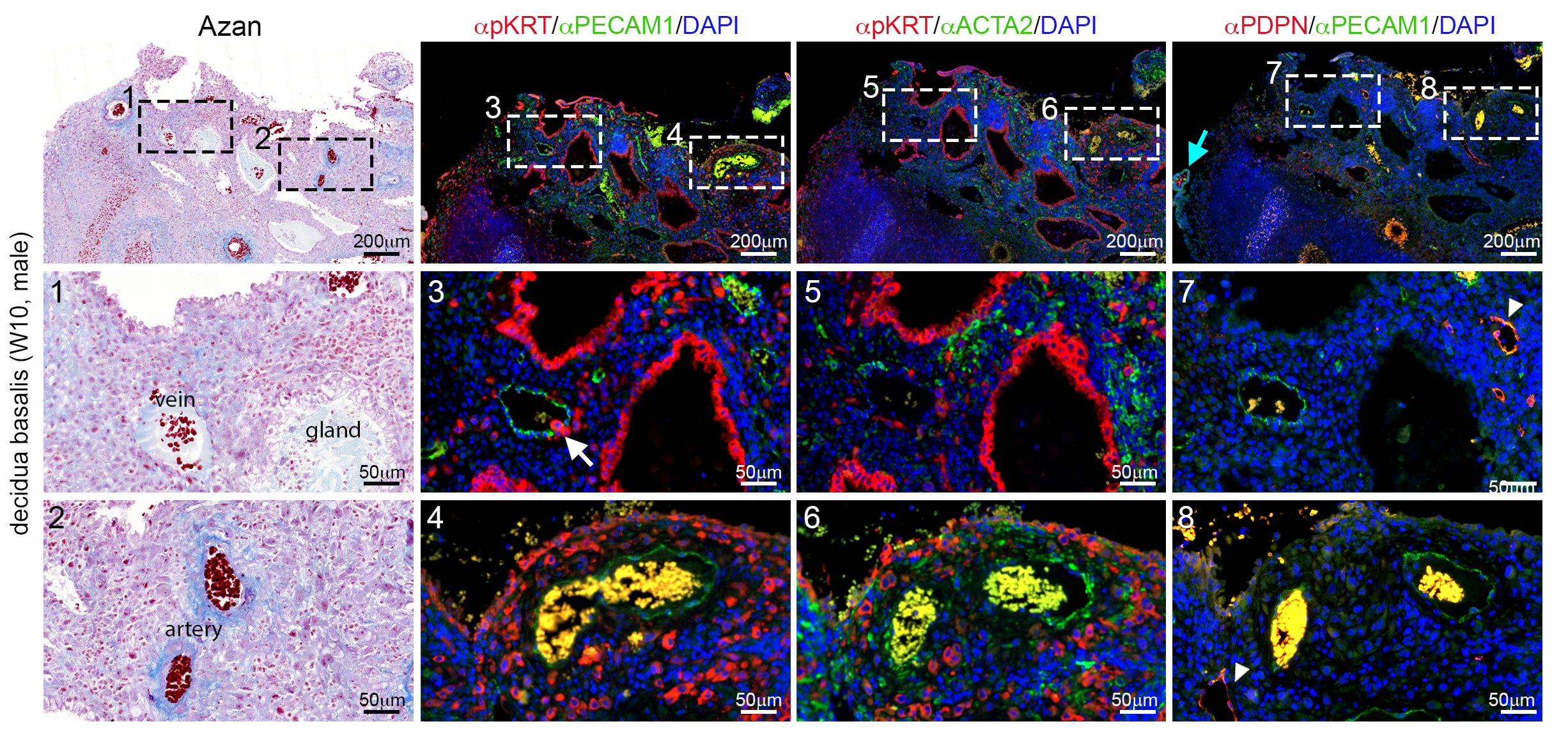

Supplement: S2 Fig — Histological sections of W10 decidua basalis showing decidual veins, arteries and lymphatic vessels. Consecutive sections were used for Azan staining (left panels), immunostained for pKRT and PECAM1 (left-middle panels), immunostained for pKRT and ACTA2 (right-middle panels) and immunostained for PDPN and PECAM1 (right panels). The middle and bottom rows show magnifications of the (numbered) dashed boxes in the top rows. White arrow depicts EVTs invading a vein; cyan arrow points to an attached placental villus; white arrowheads point to a PDPN-positive lymphatic vessel. FISH for chrX and chrY is shown in Fig 3. All scale bars are depicted. (TIF) [file pone.0169849.s002.tif]
