# Supplementary material for: Human Extravillous Trophoblasts Penetrate Decidual Veins and Lymphatics before Remodeling Spiral Arteries during Early Pregnancy
Source: PLoS One. 2017 Jan 12;12(1):e0169849. doi: 10.1371/journal.pone.0169849 (PMC5230788; doi:10.1371/journal.pone.0169849)
Supplement: S1 Table — (DOC) [file pone.0169849.s003.doc]

**S1 Table. Characteristics of the human decidua samples**.

| **Embryo ID** | **Sex** | **Age (weeks and days of gestation)** | **Decidua basalis**  **(No. samples)** | **Decidua parietalis**  **(No. samples)** |
| --- | --- | --- | --- | --- |
| EB3 | XY | W5.5 | 2 | 1 |
| EI1 | XY | W6.2 | 2 | 4 |
| EJ2 | XY | W7 | 2 | 4 |
| EM3 | XY | W7.2 | 1 | 1 |
| GP4 | XY | W7.2 | 1 | - |
| EL1 | XY | W7.5 | 1 | - |
| EC2 | XY | W8.1 | 2 | 1 |
| ED2 | XXY | W8.4 | 1 | 1 |
| FH1 | XY | W8.4 | 1 | 1 |
| GS1 | XY | W8.5 | 1 | 1 |
| EO3 | XY | W10 | 1 | 1 |
| FQ4 | XY | W10.6 | 1 | 3 |
| EG2 | XY | W12 | 3 | 2 |
| **Total** | - | - | **19** | **20** |
